# Exosomal circular RNA hsa_circ_0006220, and hsa_circ_0001666 as biomarkers in the diagnosis of pancreatic cancer

**DOI:** 10.1002/jcla.24447

**Published:** 2022-04-21

**Authors:** Lu Hong, Liu Xu, Lufei Jin, Kaiwei Xu, Weiwei Tang, Yuchao Zhu, Xuedan Qiu, Jianhua Wang

**Affiliations:** ^1^ Department of Radiology The Affiliated Hospital of Medical School of Ningbo University Ningbo China; ^2^ Ningbo Medical Center Lihuili Eastern Hospital Ningbo China

**Keywords:** biomarker, CircRNA, exosome, pancreatic cancer

## Abstract

**Background:**

Pancreatic cancer is a highly malignant tumor of the digestive system.

**Objective:**

Exosomal circular RNA can be used as a biomarker for the early diagnosis of pancreatic cancer.

**Methods:**

The expression of various differentially expressed circRNAs in pancreatic cancer tissues was analyzed by gene chip, exosome expression was verified by electron microscopy and Western blotting, and the expression of exosomal circRNA in pancreatic cancer cells, tissues, and plasma were determined by quantitative reverse transcription‐polymerase chain reaction (qRT‐PCR).

**Results:**

Compared with healthy controls, hsa_circ_0006220 and hsa_circ_0001666 were highly expressed in exosomes in the plasma of pancreatic cancer patients. The AUC values were 0.7817 for hsa_circ_0006220, 0.8062 for hsa_circ_0001666, and 0.884 for the combined diagnosis. In addition, clinicopathological features revealed that the expression of hsa_circ_0006220 in plasma exosomes from pancreatic cancer patients was associated with CA19‐9 levels (*p* = 0.0001) and lymph node metastasis (*p* = 0.0005). The expression of hsa_circ_0001666 was correlated with both tumor size (*p* = 0.0157) and CA19‐9 level (*p* = 0.0001).

**Conclusions:**

The high expression of exosomal hsa_circ_0001666 and hsa_circ_0006220 suggests that these can be used as new biomarkers for the diagnosis and treatment of pancreatic cancer.

## Graphical abstract

Pancreatic cancer is a highly malignant tumor of the digestive system. Exosomal circular RNA can be used as a biomarker for the early diagnosis of pancreatic cancer. The expression of various differentially expressed circRNAs in pancreatic cancer tissues was analyzed by gene chip, exosome expression was verified by electron microscopy and Western blotting, and the expression of exosomal circRNA in pancreatic cancer cells, tissues, and plasma were determined by quantitative reverse transcription PCR (qRT‐PCR). Exosomes hsa_circ_0001666 and hsa_circ_0006220 were highly expressed in pancreatic cancer tissues, cell lines, and plasma samples of pancreatic cancer patients and were related to clinical staging. The high expression of exosomal hsa_circ_0001666 and hsa_circ_000622 suggests that these can be used as new biomarkers for the diagnosis and treatment of pancreatic cancer.

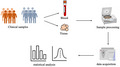

## 1 INTRODUCTION

Pancreatic cancer is a highly malignant tumor of the digestive system. In recent years, due to changes in eating habits and lifestyles, the incidence of pancreatic cancer has increased year by year to become a serious health concern.^1^ Surgical resection is an effective treatment method for pancreatic cancer. However, due to the difficulty in the early detection of pancreatic cancer and the lack of effective screening indicators, the tumor is frequently at an advanced stage at the time of diagnosis. Pancreatic cancer has one of the worst prognoses of malignant tumors with an overall 5‐year survival rate of only about 8%.^2^, ^3^ Therefore, the key to improving the survival rate of pancreatic cancer lies in the early diagnosis and early treatment. The search for relevant molecular markers for the detection of high‐risk pancreatic cancer will help patients achieve early detection and timely intervention and treatment.

Exosomes are membrane vesicles with diameters of 40–100 nm that originate from endocytosis. They are secreted by various cells and contain large amounts of nucleic acids (circRNA, lncRNA, miRNA, mRNA, and DNA), proteins, enzymes, and other substances. They are usually extracellular and play important roles in communication. Since exosomes released by cells into the body fluids show different protein and RNA contents in healthy subjects and patients with different diseases, they have the potential for being used as diagnostic markers.^4^ Tumor‐derived exosomes are rich in non‐coding RNAs that may be used as tumor markers.^5^ For example, in the plasma of gastric cancer patients, the expression of exosomes circ‐KIAA1244 was found to be correlated with the TNM staging, lymph node metastasis, and overall survival time of the patients, suggesting their use as a new circulating biomarker for gastric cancer.^6^ Therefore, this method could act like a "liquid biopsy" as a marker for the early diagnosis of pancreatic cancer.

Circular RNA (circRNA) is an RNA with a continuous covalent closed‐loop structure. CircRNAs can be divided into non‐coding and coding circRNAs according to the ability to encode protein.^7^ With the continuous development of sequencing technologies, circRNAs have been found to be both species‐specific and tissue‐specific^8^, ^9^ and are widely expressed in the cytoplasm and nucleus. In addition, they play important regulatory roles in the occurrence and development of many diseases (especially cancer). Since circRNA has a stable circular structure and no unstable 3’ and 5’ ends, the molecule is not easily degraded by nucleases, making it an ideal biomarker for disease detection.^10^ These findings also suggest that circRNA may be an ideal diagnostic marker for pancreatic cancer.

However, there are few studies on the diagnostic value of exosomal circRNAs for pancreatic cancer. In this study, we screened five expressed circRNA molecules through the GEO database, collected clinical plasma samples, and verified the expression levels of these molecules in exosomes. The experimental results showed that hsa_circ_0001666 and hsa_circ_0006220 are located in the plasma of pancreatic cancer patients. The expression of these circRNAs in the body and their diagnostic potential were analyzed by receiver operating characteristic (ROC) curves. The findings of this study will assist the development of screening tests using plasma exosomal circRNA for the diagnosis of patients with pancreatic cancer.

## 2 MATERIALS AND METHODS

### 2.1 Clinical samples

In this study, from June 2018 to June 2020, 30 cases of pancreatic cancer tissues and their corresponding paired adjacent tissues were collected in the East District Hospital of Ningbo Li Huili Hospital, China. At the same time, the plasma of 62 pancreatic cancer patients and 62 healthy subjects were collected. This study was approved by the hospital ethics committee. All patients provided written informed consent.

### 2.2 Exclusion criteria

Patients with pancreatic ductal adenocarcinoma undergoing preoperative neoadjuvant chemotherapy or radiotherapy; patients with benign or borderline malignant tumors of the pancreas; patients with pancreatic metastases (including kidney cancer and pancreatic cancer that had undergone metastasis to the pancreas); serous patients with cystadenocarcinoma or mucinous cystadenocarcinoma; patients with chronic pancreatitis that had experienced acute pancreatitis within the previous three months; patients with chronic pancreatitis with a tendency to become malignant within six months of follow‐up, and patients with blood specimens showing hemolysis of grade 5 or above.

### 2.3 Cell culture

Human embryonic kidney HEK 293Tcells, human pancreatic duct cells hTERT‐HPNE, and two human pancreatic cancer cell lines Panc2, MIA PaCa‐2 were cultured in Dulbecco's modified Eagle medium (DMEM) containing 10% fetal bovine serum in a cell culture incubator containing 5% CO_2_ at 37°C. The medium was changed every 36 h based on observed alterations in color and the growth state of the cells. Cells were passaged when confluent.

### 2.4 Isolation of exosome

The exosomes were isolated using the Total Exosome Isolation Kit (Invitrogen Life Technologies, Carlsbad, CA, USA) according to the manufacturer's instructions. Briefly, the serum was centrifuged at 2000 × *g* for 30 min to remove cells and debris. Following this, 400 μl clarified serum was transferred to a new tube and 0.2 volumes of the Total Exosome Isolation Reagent was added. The serum/reagent mixture was vortexed until homogenous and incubated at 4˚C for 30 min. After incubation, the samples were centrifuged at 10,000 × *g* for 10 min at room temperature. The supernatant was discarded and the exosome‐containing pellet at the bottom of the tube was resuspended in 200 μl phosphate‑buffered saline (PBS). The size distribution of the exosomes was confirmed using nanoparticle tracking analysis (Zetaview, Germany).

### 2.5 Electron microscopy

Freshly separated exosomal droplets (20 μl) were fixed on a copper network for 5 min for electron microscopy. The sample was stained with a 2% phosphotungstic acid solution for 5 min at room temperature. Dry filter paper was used to remove the excess liquid, and the sample was then baked under a 60‐W incandescent lamp for 3–5 min. The image was observed using a transmission electron microscope (Tecnai Spirit G2 BioTWIN, FEI).

### 2.6 Western blotting

The total protein was collected through lysis with radioimmunoprecipitation assay (RIPA) buffer supplemented with 1:100 protease inhibitors (Life Technologies, USA) and phosphatase inhibitor cocktail I and II (Sigma‐Aldrich, USA). The protein was quantified using a Bicinchoninic Acid Protein assay kit (Life Technologies) and separated on SDS polyacrylamide gels. The separated proteins were transferred to PVDF membranes (Bio‐Rad, Hercules, CA, USA), blocked overnight with 5% skimmed milk, and incubated overnight at 4°C with the appropriate primary antibodies, 1:1000 anti‐CD9 (EXOAB‐CD9A‐1, System Biosciences) and anti‐CD63 (EXOAB‐CD63A‐1, System Biosciences). After washing, the blots were incubated with an HRP‐conjugated secondary anti‐rabbit antibody at room temperature for 1 h. After washing, the immunoreactive bands were visualized using Immobilon Western HRP (Millipore; USA) and detected with FluorChem HD2 (Proteinsimple; USA).

### 2.7 RNA extraction and qRT‐PCR

The RNA was extracted from the tissue samples using TRIzol (Invitrogen, USA), following the manufacturer's instructions. TRIzol LS (Invitrogen) was used to isolate total RNA from the plasma. A Prime Script TM II cDNA Synthesis Kit (TaKaRa, Japan) was employed to reverse‐transcribe 1 µg total RNA to cDNA. qRT‐PCR (Roche, Switzerland) combined with SYBR^®^ GreenⅠmethod (Roche, Switzerland) was used for amplification. The reaction conditions used were as follows: pre‐denaturation at 95°C for 30 s, PCR reaction at 95°C for 5 s, 60°C for 20 s, for 40 cycles, and annealing at 50°C for 30 s. The 2^−△△ct^ method was applied to determine the relative circRNA expression.

### 2.8 Statistical analysis

SPSS 23.0 and GraphPad 8.0 were used to perform statistical analysis on all the experimental data (each experiment was repeated at least three times). In addition, the count data used cases (percentage) to indicate the potential expression of circRNA (2^−Δ△ct^ value) in pancreatic cancer and corresponding adjacent tissues, and paired t‐tests were used to determine differences. The expression of circRNA in the blood of pancreatic cancer patients and healthy subjects was determined by non‐parametric tests. Fisher's exact test and Spearman correlation coefficient were used to analyze the correlation between circRNA expression and clinicopathological parameters. Receiver operating characteristic (ROC) curves were used to evaluate the diagnostic efficiency of circRNA in pancreatic cancer samples. The Youden index was used to calculate the critical value, and the Z test was used to compare the area under the curve (AUC). *p* < 0.05 was considered statistically significant.

## 3 RESULTS

### 3.1 hsa_circ_0000977, hsa_circ_0006220, and hsa_circ_0001666 as candidate biomarkers for pancreatic cancer

**FIGURE 1:**
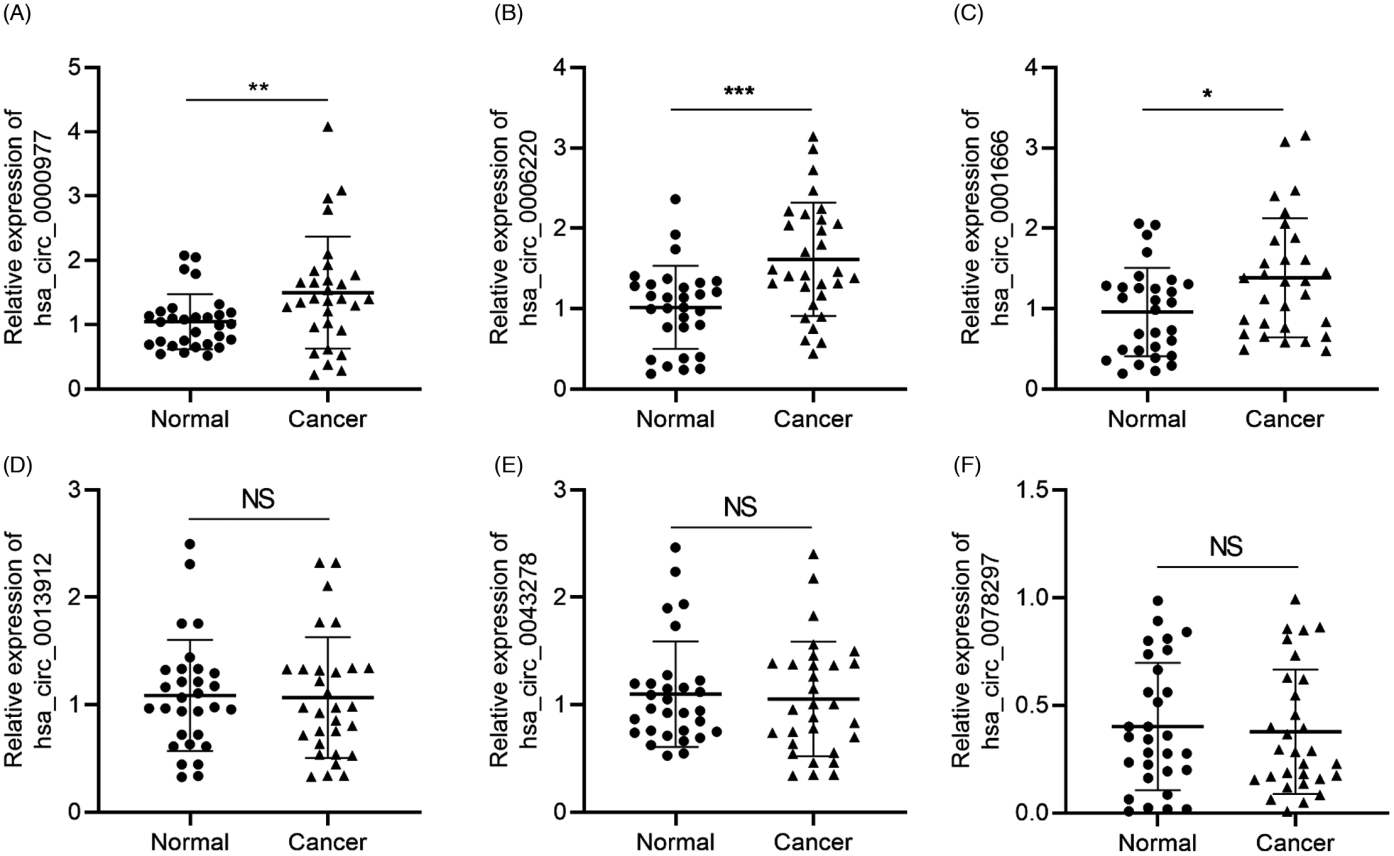
Identification of circRNAs associated with pancreatic cancer. qRT‐PCR analysis of hsa_circRNA in 30 pancreatic cancer tissues and adjacent tissues. (A) hsa_circ_ 0000977, (B) hsa_circ_0006220, (C) hsa_circ_0001666, (D) hsa_circ_0013912, (E) hsa_circ_0043278, and (F) hsa_circ_0078297

We extracted pancreatic cancer circRNA chip data (Accession No. GSE43796) according to the GEO database and screened out the six most significantly upregulated circRNAs in pancreatic cancer chips, namely hsa_circ_0000977, hsa_circ_0006220, hsa_circ_0001666, hsa_circ_0013912, hsa_circ_0043278, and hsa_circ_0078297. qRT‐PCR was used for detection, and analysis of pancreatic cancer tissue samples (*n* = 30) found that hsa_circ_0000977 (Figure 1A), hsa_circ_0006220 (Figure 1B), and hsa_circ_0001666 (Figure 1C) were significantly higher in pancreatic cancer tissues than in their counterpart adjacent tissues. The expression of hsa_circ_0013912 (Figure 1D), hsa_circ_0043278 (Figure 1E), and hsa_circ_0078297 (Figure 1F) did not differ significantly between pancreatic cancer and adjacent tissues.

### 3.2 Exosomes are abundantly enriched in plasma samples

**FIGURE 2:**
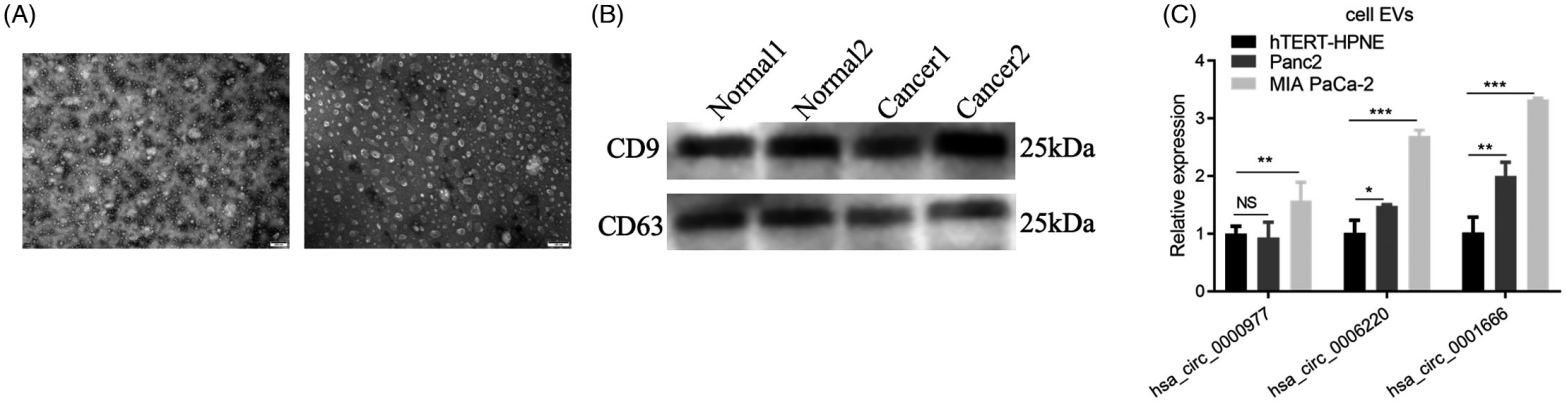
Detection and identification of plasma exosomes. (A) Electron micrographs of plasma exosomes; (B) Western blot showing CD9 and CD63; (C) hsa_circ_0000977, hsa_circ_0006220, and hsa_circ_0001666 expression in exosomes

Many studies have confirmed that circRNA is stably enriched in tumor exosomes. The transfer of nucleic acids such as circRNA by exosomes protects nucleic acids from degradation and dilution in the extracellular space, allowing the passage of circRNAs through tissues or the blood. Exosomal circRNAs have great potential as molecular markers for early tumor diagnosis. Therefore, we extracted exosomes from the plasma of patients with pancreatic cancer and photographed them under transmission electron microscopy. The results showed that the exosomes derived from plasma were 80–120 nm in size with the appearance of small vesicles with membranous structures. The exosomal characteristics of the samples were verified by electron microscopy. Western blotting was used to determine the expression of the exosomal marker proteins CD9 and CD63 to determine the success of the exosome isolation and purification procedures. The results showed that CD9 and CD63 were present in the plasma exosomes (Figure 2B), indicating that the exosomes were successfully extracted. At the same time, we detected the relative expression of hsa_circ_0000977, hsa_circ_0006220, and hsa_circ_0001666 in hTERT‐HPNE (human pancreatic duct cells) and two human pancreatic cancer cell lines (Panc2, MIA PaCa‐2) exosomes by qRT‐PCR. The results showed that compared with normal cells, hsa_circ_0000977, hsa_circ_0006220, and hsa_circ_0001666 were expressed at significantly higher levels in the two pancreatic cancer cell lines (Figure 2C) while hsa_circ_0000977, hsa_circ_0006220, and hsa_circ_0001666 were all up‐regulated in pancreatic cancer tissues and exosomes, indicating that hsa_circ_0000977, hsa_circ_0006220, and hsa_circ_0001666 were candidate circRNAs for the subsequent studies.

### 3.3 hsa_circ_0006220 and hsa_circ_0001666 are highly expressed in plasma exosomes of patients with pancreatic cancer

**FIGURE 3:**
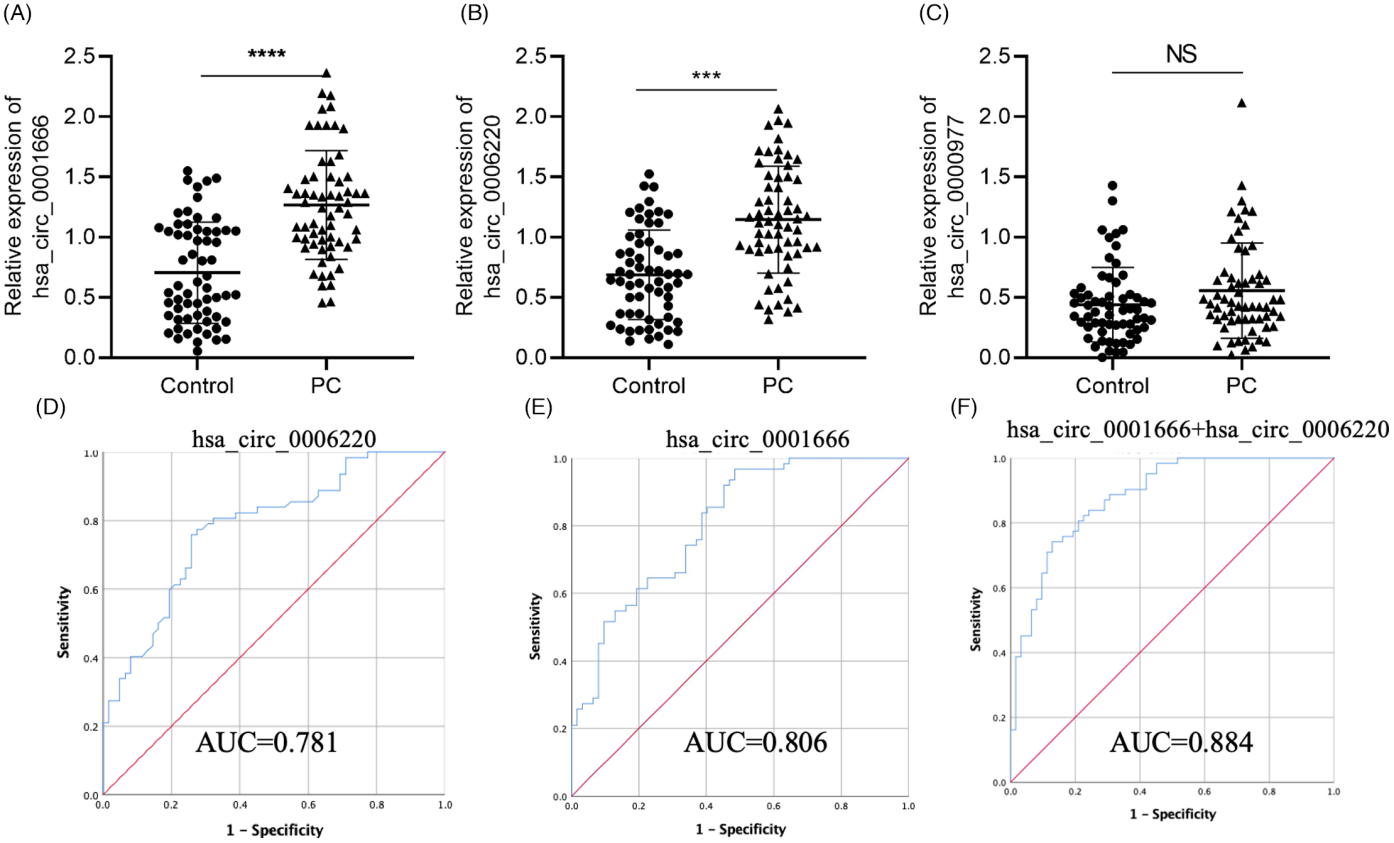
CircRNA expression in plasma exosomes. qRT‐PCR analysis of (A) circ_0000977, (B) hsa_circ_0006220, and (C) hsa_circ_0001666 in the plasma exudates of 62 patients with pancreatic cancer and healthy individuals; (D) diagnostic value of hsa_circ_0006220 in patients with pancreatic cancer; (E) diagnostic value of hsa_circ_0001666 in patients with pancreatic cancer; (F) diagnostic value of the combination of hsa_circ_0006220 and hsa_circ_0001666 in patients with pancreatic cancer

The three selected circRNA candidates were further verified in plasma exosomal samples. These samples included 62 samples from patients with pancreatic cancer and 62 plasma exosomal samples from healthy subjects. The qRT‐PCR results showed that there was no statistical difference in the expression of hsa_circ_0000977 in plasma exosomes of pancreatic cancer patients compared with healthy controls (Figure 3A). In contrast, hsa_circ_0006220 (Figure 3B) and hsa_circ_0001666 (Figure 3C) showed significantly higher expression in the exosomes of patients with pancreatic cancer.

We evaluated the ability of hsa_circ_0006220 and hsa_circ_0001666 to distinguish between normal healthy people and pancreatic cancer through the AUC of the ROC curve. The hsa_circ_0006220 AUC value was 0.7817 (95% CI = 70.17% to 86.16%, with a sensitivity = 77.42% and a specificity = 72.58%, Figure 3D); the hsa_circ_0001666 AUC value was 0.8062 (95% CI = 73.16% to 88.08%, sensitivity = 96.77%, specificity = 51.61%, Figure 3E). The results showed that hsa_circ_0006220 and hsa_circ_0001666 could effectively distinguish between the normal control group and pancreatic cancer patients. Among them, hsa_circ_0001666 had the highest AUC value, suggesting a good diagnostic value, while hsa_circ_0006220 had a somewhat lower diagnostic value.

Evidence shows that a combination of tumor markers can improve the accuracy of diagnosis. In this study, we combined hsa_circ_0006220 and hsa_circ_0001666. The results showed that in distinguishing normal people from pancreatic cancer patients, The AUC value of the combination was 0.884 (95% CI = 82.7% to 94.1%, with a sensitivity = 0.742% and a specificity = 0.871%, Figure 3F), which was significantly higher than that of a single molecule. This indicated that the combination of hsa_circ_0006220 and hsa_circ_0001666 had improved diagnostic value and high accuracy.

The above data showed that the expression of hsa_circ_0006220 and hsa_circ_0001666 in plasma exosomes samples of pancreatic cancer was significantly upregulated. Therefore, we analyzed the association between hsa_circ_0006220 and hsa_circ_0001666 and clinicopathological factors in patients with pancreatic cancer. As shown in Table 1, the expression of hsa_circ_0006220 in plasma exosomes from pancreatic cancer patients was closely related to CA19‐9 (*p* = 0.0001) and lymph node metastasis (*p* = 0.0005). However, there was no significant correlation between the expression of hsa_circ_0006220 and other clinicopathological factors, including age (*p* = 0.0730), sex (*p* = 0.3074), tumor size (*p* = 0.4208), clinical stage (*p* = 0.0384), and degree of differentiation (*p* = 0.0684). As shown in Table 2, the expression of hsa_circ_0001666 in the plasma exosomes of pancreatic cancer patients was closely related to tumor size (*p* = 0.0157) and CA19‐9 (*p* = 0.0001). However, there was no significant correlation between hsa_circ_0001666 expression and other clinicopathological factors, including age (*p* = 0.2003), sex (*p* = 0.6098), lymph node metastasis (*p* = 0.2766), clinical stage (*p* = 02003), and degree of differentiation (*p* = 0.2244).

**TABLE 1:** Clinicopathological factors of plasma exosomes samples of patients with pancreatic cancer and expression of hsa_circ_0006220

Characteristics	Case no.	hsa_circ_0006220 level		*p* value
		Low	High	
Total cases	62	31	31	
Gender				
Male	34	19	15	0.3074
Female	28	12	16	
Age				
≤60	27	10	17	0.0730
>60	35	21	14	
Tumor size(cm)				
≤4	41	19	22	0.4208
>4	21	12	9	
CA19‐9				
Positive	33	9	24	0.0001
Negative	29	22	7	
Clinical stage				
Ⅰ/Ⅱ	27	13	14	0.7978
Ⅲ/Ⅳ	35	18	17	
Differentiated degree				
Low and middle	48	21	27	0.0684
High	14	10	4	
Lymph node metastasis				
No	46	17	29	0.0005
Yes	16	14	2	

**TABLE 2:** Clinicopathological factors of plasma exosomes samples of patients with pancreatic cancer and expression of hsa_circ_0001666

Characteristics	Case no.	hsa_circ_0001666 level		*p* value
		Low	High	
Total cases	62	31	31	
Gender				
Male	34	16	18	0.6098
Female	28	15	13	
Age				
≤60	27	16	11	0.2003
>60	35	15	20	
Tumor size(cm)				
≤4	41	25	16	0.0157
>4	21	6	15	
CA19‐9				
Positive	33	9	24	0.0001
Negative	29	22	7	
Clinical stage				
Ⅰ/Ⅱ	27	16	11	0.2003
Ⅲ/Ⅳ	35	15	20	
Differentiated degree				
Low and middle	48	22	26	0.2244
High	14	9	5	
Lymph node metastasis				
No	46	24	21	0.2766
Yes	16	6	10	

### 3.4 Stability of hsa_circ_0006220 and hsa_circ_0001666

**FIGURE 4:**
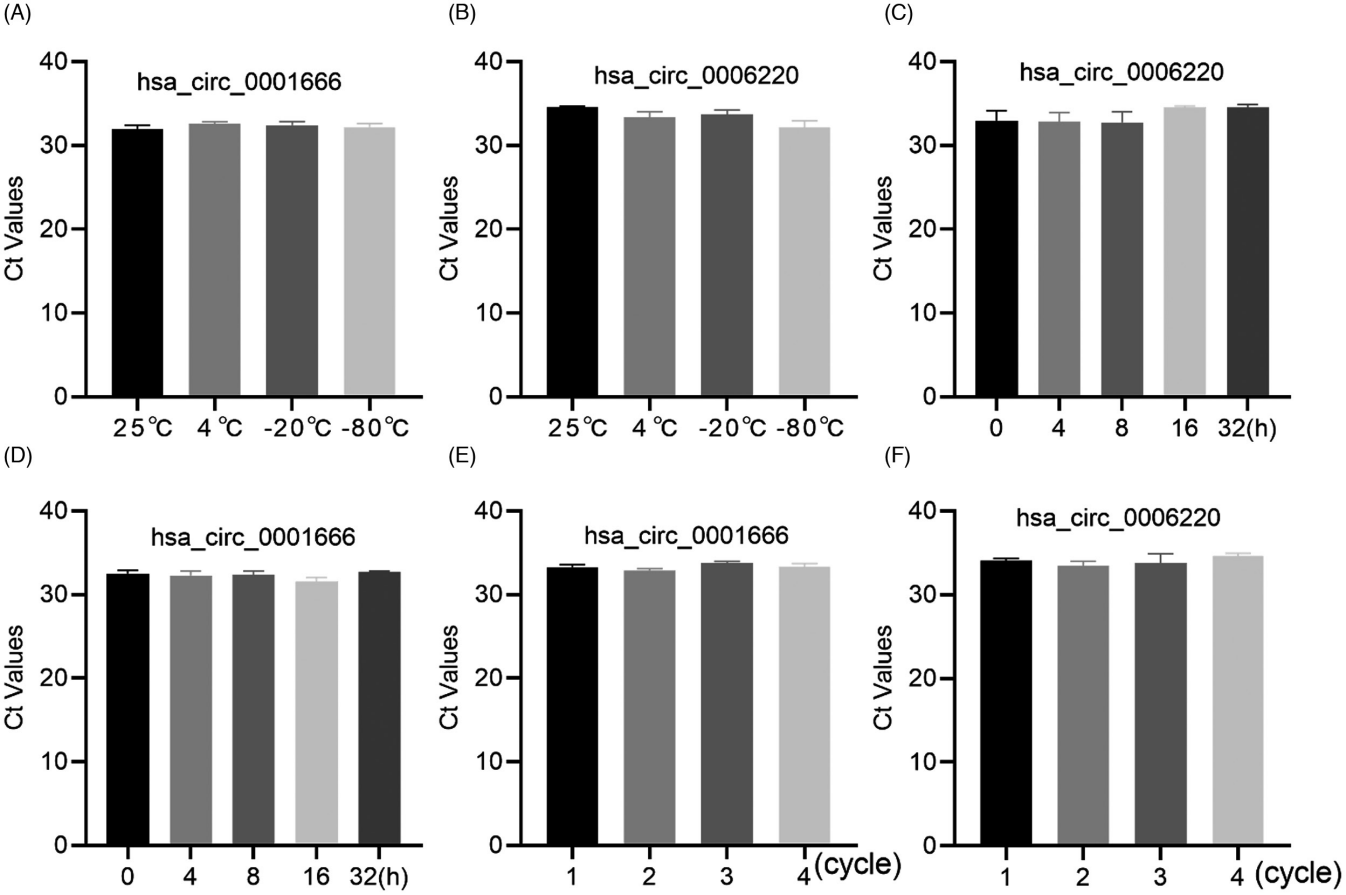
Stability of hsa_circ_0001666 and hsa_circ_0006220. Expression of hsa_circ_0001666 and hsa_circ_0006220 was detected by qRT‐PCR at different storage temperatures (A–B), (C–D) repeated freeze‐thaw cycles, and storage times (E–F)

Generally, storage temperature, freeze‐thaw cycles, and time are the main problems encountered in clinical blood sample testing. Therefore, in order to study the stability of hsa_circ_0006220 and hsa_circ_0001666 in plasma exosomes, fresh plasma samples from three patients with pancreatic cancer were divided into equal parts and stored at different temperatures (25, 4, −20, and −80°C), stored for 24 h, or exposed to different incubation times (4, 8, 16, and 32 h) at room temperature, or treated with 1, 2, 3, or 4 repeated freeze‐thaw cycles. The results showed that the relative expression levels of hsa_circ_0006220 and hsa_circ_0001666 were not statistically different at different temperatures (Figure 4A, B), different incubation times (Figure 4C, D), and after repeated freeze‐thaw cycles (Figure 4E, F). Therefore, the stability of hsa_circ_0006220 and hsa_circ_0001666 indicates that they may become new diagnostic markers for pancreatic cancer.

## 4 DISCUSSION

Pancreatic cancer is one of the most malignant tumors of the digestive system. Although the prognosis has improved slightly recently, the 5‐year survival rate is still less than 8%. Its incidence rate is still on the rise all over the world, and the case fatality rate is expected to rank second among all malignant tumors in Western countries in 2030.^11^, ^12^ At the time of diagnosis, about 80–85% of patients have unresectable or metastatic pancreatic cancer.^13^ Even if a small percentage of patients are diagnosed with locally resectable tumors, the prognosis is still very poor, and only about 20% of patients survive five years after surgery. In the past ten years, advances in the diagnosis methods, perioperative management, radiotherapy technology, and systemic therapy for advanced pancreatic cancer patients have made some progress, but the treatment of pancreatic cancer is still very limited. There is an urgent need to find new methods for screening high‐risk patients to detect pancreatic tumors at an early stage, which will have far‐reaching significance for the clinical treatment of pancreatic cancer.

At present, the most commonly used method for preoperative diagnosis of pancreatic cancer is imaging examination combined with the tumor markers CEA and CA19‐9.^1^, ^14^, ^15^ Commonly used clinical imaging methods for pancreatic cancer include ultrasound, CT, MRI, and endoscopic ultrasound. All these methods have difficulty in diagnosing early pancreatic cancer with tumor diameters of <1 cm. The specificity of CA19‐9 in pancreatic cancer screening is about 70%, but this improvement is not obvious in early‐stage patients. At the same time, about 5–10% of pancreatic cancer patients do not express CA19‐9 and for CEA, the average specificity is 79%, and the average sensitivity is 54%.^16^ Studies have shown significantly higher activity of class III alcohol dehydrogenase (ADH) in pancreatic cancer tissue and plasma in comparison with healthy tissue and serum. In addition, the assessment of the diagnostic ability of ADH III showed a diagnostic sensitivity and specificity of 70% and 76%, respectively, and an area under the ROC curve of 0.64. ADH III thus has potential value in the diagnosis of pancreatic cancer. However,^17^, ^18^, ^19^ these tumor markers are not currently recommended for screening and diagnosis of early pancreatic cancer.^20^ Therefore, it is urgent to find new diagnostic markers or combinations to improve the sensitivity and specificity for the early diagnosis of pancreatic cancer.

Current research shows that circRNA is enriched in exosomes compared with parental cells. Recently, exosomal circRNA has received more attention as a biomarker and has been shown to have a significant impact on pathophysiological progress.^5^ At the same time, the enrichment and stability of circRNA in exosomes suggest its potential as a disease marker. Our previous study showed that hsa_circ_0013587 is highly expressed in the tissues, cells, and plasma of patients with pancreatic cancer, indicating its potential as a new diagnostic biomarker for pancreatic cancer.^21^ In addition, the role of exosomes circRNA circ‐PDE8A in regulating the invasion of pancreatic cancer through the miR‐338/MACC1/MET pathway has also been described.^22^ Nevertheless, there are still few studies on exosomal circRNA in the diagnosis of pancreatic cancer. In our study, we extracted six differentially expressed circRNAs from GEO data and successfully verified the existence of exosomes hsa_circ_0000977, hsa_circ_0006220, and hsa_circ_0001666 in the plasma. Of these, hsa_circ_0001666 and hsa_circ_0006220 showed significantly higher expression in the plasma of pancreatic cancer patients. However, there has not been much research on the two molecules hsa_circ_0001666 and hsa_circ_0006220.

Numerous studies have shown that exosomal circRNAs in the plasma or serum can be used as diagnostic biomarkers for cancer. For example, circ‐KIAA1244 in plasma is released in the form of exosomes, which can be used as a new circulating biomarker for gastric cancer detection. This was found to have a sensitivity of 77.42 and a specificity of 68.00% in the screening of gastric cancer patients and normal individuals^6^; circulating exosomal hsa_circ_0004771 is related to the TNM staging of colorectal cancer and cancer metastasis. The AUC that distinguished colorectal cancer patients at all stages from healthy people was 0.88 (95% CI, 0.815–0.940), with a sensitivity of 80.91%, and a specificity of 82.86%.^23^ Exosomal circ_0070396 was found to have significant diagnostic value in distinguishing between non‐cancerous control, chronic hepatitis B, liver cirrhosis, and liver cancer patients. Among them, the diagnostic combination of exosomes circ_0070396 + AFP could distinguish between the non‐cancerous control and liver cancer with 81.98% sensitivity and 100% specificity.^24^ In addition. exosomal circ‐PDE8A derived from pancreatic cancer cells was found to be able to enter the blood circulation and was related to the progression and prognosis of pancreatic cancer.^22^ In our study, the exosomal hsa_circ_0001666 and hsa_circ_0006220 performed well in distinguishing pancreatic cancer patients from healthy subjects, with a higher diagnostic efficacy when used in combination. In addition, hsa_circ_0001666 and hsa_circ_0006220 also have value in guiding the diagnosis of pancreatic cancer and helping to determine the tumor stage. However, the independent and combined diagnostic value of hsa_circ_0001666 and hsa_circ_0006220 require further optimization using clinical large‐scale cohort, multi‐sample, prospective, and multi‐center studies.

At the same time, exosomal circRNAs show good diagnostic performance, not only because of their high specificity and sensitivity but also because of their structure and stability in the blood.^22^ We observed through RNase R and ActD that the expression of exosomal circRNA was not significantly affected, and neither long‐term storage at room temperature nor repeated freezing and thawing altered the expression level of the exosomal circRNAs.

Recently, several studies have reported that exosomal circRNA mediates the progression of pancreatic cancer through various mechanisms such as promoting the invasive growth of cells, regulating the permeability of the endothelial monolayer, promoting metastasis, and enhancing glycolysis to promote drug resistance.^22^, ^25^, ^26^, ^27^ In this study, the exosomes hsa_circ_0001666 and hsa_circ_0006220 were found to be effective for pancreatic cancer screening and were shown to be related to the cancer stage, suggesting that they may be involved in the occurrence and development of pancreatic cancer. This mechanism remains to be further explored.

## CONFLICT OF INTEREST

All authors declare no conflicts of interest.

## AUTHOR CONTRIBUTIONS

LH, LX, and YCZ contributed to the experimental design. LFJ, LX, WWT, and XDQ contributed to clinical diagnosis and sample collection. LH, KWX, and LFJ contributed to testing. JHW and LH contributed to the data analysis, result interpretation, and writing. All authors have reviewed and approved the final study.

## Data Availability

The data used in the current study are available from the corresponding author upon reasonable request.
